# Molecular Network of Colorectal Cancer and Current Therapeutic Options

**DOI:** 10.3389/fonc.2022.852927

**Published:** 2022-04-06

**Authors:** Zhe Huang, Mingli Yang

**Affiliations:** ^1^The Department of 11^th^ General Surgery, Minimally Invasive Colorectal Hernia Unit, Shengjing Hospital of China Medical University, Shenyang, China; ^2^The Department of 3Oncology, Gastrointestinal Cancer Unit, Shengjing Hospital of China Medical University, Shenyang, China

**Keywords:** colorectal cancer, cellular signaling, microenvironment modulation, epigenetic changes, biomarkers

## Abstract

Colorectal cancer (CRC), a leading cause of cancer-related mortalities globally, results from the accumulation of multiple genetic and epigenetic alterations in the normal colonic and rectum epithelium, leading to the progression from colorectal adenomas to invasive carcinomas. Almost half of CRC patients will develop metastases in the course of the disease and most patients with metastatic CRC are incurable. Particularly, the 5-year survival rate of patients with stage 4 CRC at diagnosis is less than 10%. Although genetic understanding of these CRC tumors and paired metastases has led to major advances in elucidating early driver genes responsible for carcinogenesis and metastasis, the pathophysiological contribution of transcriptional and epigenetic aberrations in this malignancy which influence many central signaling pathways have attracted attention recently. Therefore, treatments that could affect several different molecular pathways may have pivotal implications for their efficacy. In this review, we summarize our current knowledge on the molecular network of CRC, including cellular signaling pathways, CRC microenvironment modulation, epigenetic changes, and CRC biomarkers for diagnosis and predictive/prognostic use. We also provide an overview of opportunities for the treatment and prevention strategies in this field.

## Introduction

Colorectal cancer (CRC) is the third diagnosed malignant tumor worldwide, accounting for approximately 9% of annual cancer death (1). More than 90% of CRCs are adenocarcinoma, resulting from normal glandular colonic and rectum epithelium. Other rare types include carcinoid tumors, gastrointestinal stromal tumors, colorectal lymphoma, squamous cell carcinomas, leiomyosarcomas, and melanomas (2). Approximately 65% of CRC cases developed sporadically, without a family history or inherited genetic mutations predisposition, through multiple acquired somatic genomic and epigenetic alterations (3, 4). Other cases are associated with heritable components such as family history (25%), hereditary cancer syndrome (5%), some known CRCs low-penetrance genetic variations (<1%), and other unknown inherited genomic alterations (4, 5).

**Figure 1:**
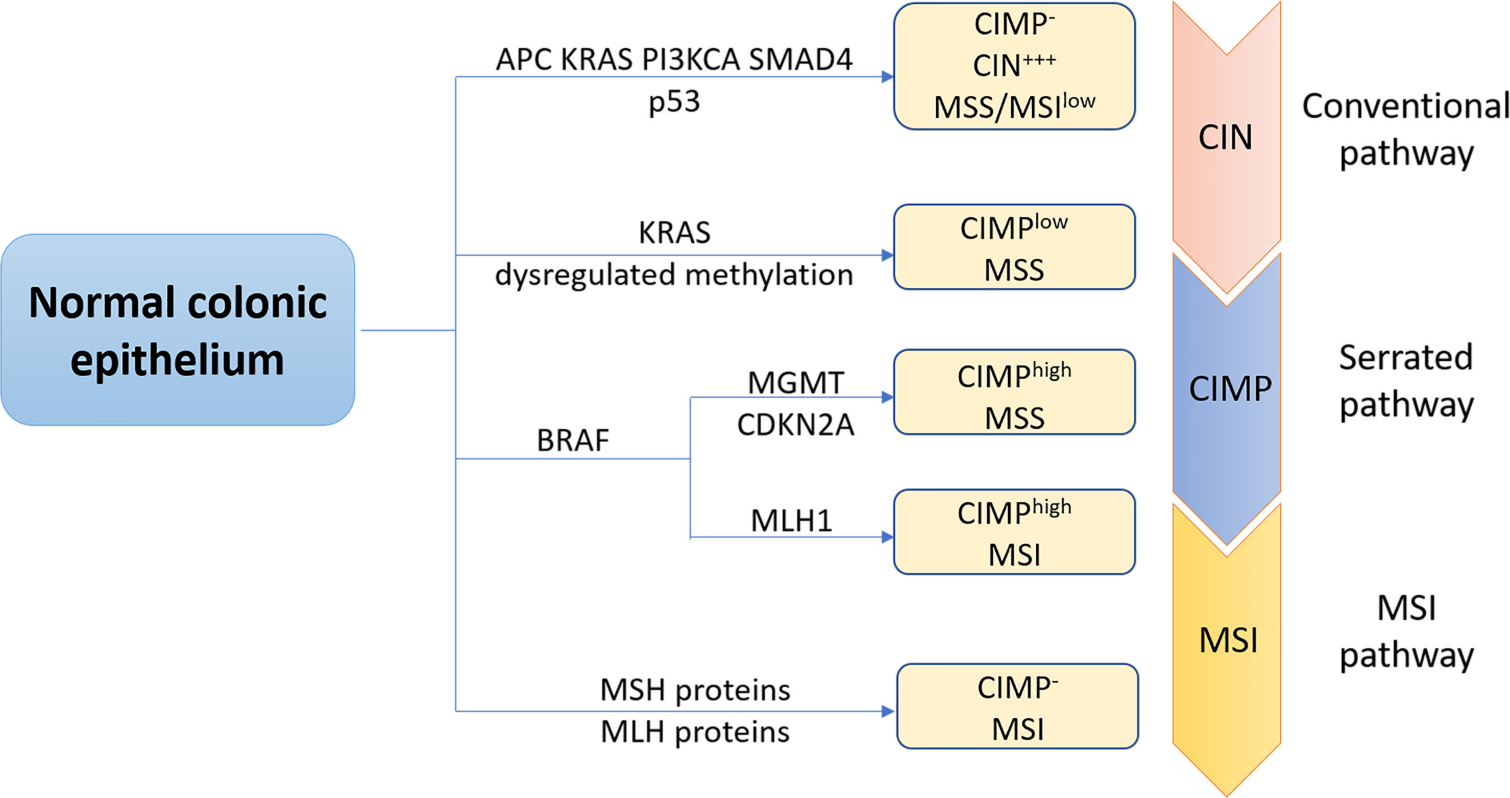
The three major molecular pathways of colorectal cancer. The conventional chromosomal instability (CIN) pathway, initiated by APC mutation, then followed by mutations in KRAS, PIK3CA and SMAD4, loss of heterozygosity of p53 mutation was observed in most CRC cases. CRC progress and development via this pathway is often associated with no or low levels of the CpG island methylation pathway (CIMP- ), high levels of CIN (CIW^+++^), and microsatellite stability (MSS). Approximately one third CRC cases is regulated through the serrated pathway, which can be subdivided into CIMP^low^ MSS tumors with KRAS mutations, BRAF mutant CIMP^high^ MSS tumors or BRAF mutant CIMP^high^ microsatellite instability (MSI) tumors. Serrated pathway is commonly associated with silencing of 0-6-methylguanine-DNA methyltransferase (MGMT), cyclin-dependent kinase inhibitor 2A (CDKN2A) or MLHl. MSI pathway is a third important pathway of CRC caused by dysfunction of DNA mismatch repair genes, encoding Mutl homolog (MLH) or MutS homolog (MSH) proteins.

At the molecular level, CRC, like other solid tumors, is a heterogeneous disease. This can be attributed to at least three major molecular pathways (**Figure 1**). The most common pathway is the chromosomal instability (CIN), occurring in 85% of sporadic CRC (sCRC), characterized by chromosome structure and number abnormalities, frequent loss of heterozygosity (LOH) at tumor suppressor gene loci, gain or loss of chromosomal segments and chromosomal rearrangements, further resulting in gene copy number variations (6). These alterations typically are associated with mutations in specific oncogenes or tumor suppressors genes such as adenomatous polyposis coli (*APC*), Kirsten rat sarcoma virus (*KRAS*), phosphatidylinositol 4,5-bisphosphate 3-kinase catalytic subunit-α (*PIK3CA*), b-raf proto-oncogene (*BRAF*), SMAD family member 4 (*SMAD4*) or *p53* (7), which regulate cell proliferation and cell cycle and play pivotal roles in CRC initiation and progression pathways. Another important pathway to CRC is the microsatellite instability (MSI), caused by dysfunction of DNA mismatch repair (MMR) genes during DNA recombination, DNA replication and DNA damage, which encodes MutL Homolog (MLH) proteins or MutS homolog (MSH) proteins. Therefore, it is often associated with genetic hypermutability (8). CpG island methylator phenotype (CIMP) comprises the third major pathway to CRC. CIMP positive tumors can be divided into CIMP^high^ tumors, with BRAF mutations, MLH1 methylation, and silencing of O-6-methylguanine-DNA methyltransferase (MGMT) or cyclin-dependent kinase inhibitor 2A (CDKN2A) and CIMP^low^ tumors, with KRAS mutations. In fact, CRC pathogenesis is normally associated with multiple pathways. Specifically, approximately one third CRC cases develop *via* a serrated pathway that is associated with either KRAS or BRAF mutations in addition to CIMP mutations (CIMP^low^ or CIMP^high^).

Generally, CRC tumorigenesis initiates with the transformation of normal colorectal epithelial cells by a spontaneous mutation, environmental mutagens, genetic or epigenetic alterations. Then, these initiated cells rapidly expanded to form aberrant crypt foci and early adenoma, driven by mutations that cause hyperproliferation, such as APC mutations, or other signaling pathways such as WNT-β-catenin, cytokines, chemokines, and growth factors from the tumor microenvironment (TME) (9). Thus, mutations such as SMAD4, cell division control protein 4 (CDC4) and transforming growth beta factor 2 (TGFBR2) as well as chromosomal aberration such as LOH 18q further lead to the outgrowth of these clones into late adenoma and malignant tumors, known as tumor promotion. Further mutations (p53 and Bcl-2 Associated X-protein (BAX)), pro-angiogenic factors, extracellular matrix-degrading factors and other factors which promote CRC cells invasive motility facilitate these tumors efficiently to metastasize to distant organs and tissues, known as tumor progression.

Although genetic understanding of these CRC tumors and paired metastases has led to major advances in elucidating early driver genes responsible for carcinogenesis and metastasis, the pathophysiological contribution of transcriptional and epigenetic alterations in this malignancy influences many central signaling pathways have attracted attention recently. Therefore, the present review summarized the current knowledge on the molecular network of CRC, including cellular signaling pathways, CRC microenvironment modulation, epigenetic changes, CRC and inflammatory bowel disease (IBD), and CRC biomarkers for early diagnosis and prognostic/predictive use and provide an overview of opportunities for CRC treatment.

## Molecular Network of CRC

### Cellular Signaling Pathways

As mentioned, CRC development is a complicated multistage process with sequential mutations involved. Several cellular signaling pathways that regulate cell proliferation, differentiation, apoptosis, and survival are involved in CRC onsets, such as epidermal growth factor receptor (EGFR)/mitogen-activated protein kinase (MAPK), Wingless-related integration site (Wnt)/β-catenin, phosphoinositide 3-kinase (PI3K), transforming growth factor-β (TGF)-β, Neurogenic locus notch homolog protein (Notch), and nuclear factor (NF)-κB (10).

#### EGFR/MAPK Signaling Pathway

EGFR, a catalytic receptor tyrosine kinase (RTK), is a transmembrane protein containing an extracellular ligand-binding domain. After ligand binding, EGFR is activated and dimerized, resulting in the autophosphorylation of several tyrosine residues in the intercellular domain. Furthermore, the EGFR adaptor protein complex comprising the growth factor receptor-bound protein 2 (Grb2) and the son of sevenless (SOS) activates rat sarcoma virus (RAS) by conversion of guanosine diphosphate (GDP) to guanosine triphosphate (GTP) through its binding to phosphorylated tyrosine residues. Once RAS is activated, a kinase cascade includes mitogen-activated protein kinase kinase kinase-Raf (MAPKKK), mitogen-activated protein kinase kinase-MEK (MAPKK), MAPK and extracellular signal−regulated kinase (ERK) are initiated through phosphorylation (11). It has been reported that ERK signaling pathway regulates cell proliferation, differentiation, and survival. Dysregulated EGFR/MAPK signaling pathway has been reported in a variety of human cancers mainly because it can lead to malignant transformation and tumor progression through increased cell proliferation, prolonged survival, angiogenesis, anti-apoptosis, invasion, and metastasis (12). Previous studies have found that EGFR/MAPK signaling pathway was directly related to the CRC oncogenic processes and played crucial roles in CRC tumor growth and disease progression (13). Therefore, this pathway and its downstream signaling cascades have been reported as targets for CRC therapeutic intervention (14, 15).

#### Wnt/β-Catenin Pathway

It is known that all 19 glycoproteins from the Wnt family play regulatory roles in many developmental and carcinogenesis processes such as cell proliferation, migration, and division. In addition, Wnt/β-catenin signaling plays important role in tissue maintenance and hair, skin, and intestine regeneration. Mutations of this signaling pathway are frequently observed in sCRC. When the Wnt ligand is secreted and accumulated, it binds to its Frizzled (Fz) receptors. Then, the multifunctional glycogen synthase kinase (GSK)-3β is inactivated and β-catenin, that acts as the E-cadherin cell-cell adhesion protein and as a transcriptional activator, is stabilized, accumulated, and translocated into the nucleus where it couples with the lymphoid enhancer factor (LEF) or T-cell transcription factor (TCF) and activates specific target genes involved in proliferation and transmission. In the absence of the Wnt signal, β-catenin is targeted by casein kinase 1 (CK1) and the APC-core proteins Axin-GSK-3β complex for ubiquitination and proteasomal degradation through its phosphorylation. Wnt signaling hyperactivation contributes to tumor cell proliferation and its activation is required for tumor growth in the advanced cancer stage, especially in CRC (10, 16).

#### PI3K Signaling Pathway

PI3K is an important intracellular lipid kinase that regulates a variety of cellular activities such as cell growth, proliferation, differentiation, migration and survival (17). PI3K is a heterodimeric molecule containing two subunits, p85 (a regulatory subunit) and p110 (a catalytic subunit). Protein kinase B (AKT/PKB), a serine/threonine-protein kinase (Ser/Thr kinase) and a downstream effector of PI3K, regulates the PI3K effects on tumor growth and progression (18). AKT phosphorylation was reported to be involved in cell proliferation and apoptosis inhibition from human CRC. Therefore, inhibition of PI3K/Akt pathway was used for treatment in many cancers (19). PI3K is activated upon ligand binding to receptor tyrosine kinases (RTK). Then, activated PI3K phosphorylates phosphatidylinositol 4,5-bisphosphate (PIP2) to phosphatidylinositol 3,4,5-trisphosphate (PIP3). Next, PIP3 activates AKT upon its serine and threonine residues, resulting in cell proliferation and cell survival. AKT regulates downstream proteins such as the mammalian target of rapamycin (mTOR), which mediates cell cycle, proliferation, angiogenesis, protein translation, and growth and survival. Phosphatase and tensin homolog (PTEN), a tumor suppressor and a PI3K pathway downregulatory protein, dephosphorylates PIP3. This altered expression of the pathway is often observed in CRC which results in the continuous growth of cells and leads to cancer. Overall, PI3K signaling pathway was reported to play an oncogenic role in the initiation and development of CRC.

#### TGF-β Signaling Pathway

TGF-β signaling pathway is known to be involved in cell proliferation, growth, differentiation, division, migration and adhesion. TGF-β signaling is initiated upon its ligand binding to its receptors where these 2 heterodimer receptors group together to form a complex through receptor dimerization. Next, the kinase domain of these receptors is activated by phosphorylation and the downstream transcription factors, SMAD proteins, are further activated. Specifically, SMAD2 and SMAD3 are activated by forming phosphorylated heterodimers and this heterodimer complex binds to SMAD4 to form a heterotrimer. Then, the heterotrimers translocate into the nucleus to bind to TGF-β target genes and to regulate the transcription. Recent studies have reported that TGF-β, as a tumor suppressor, mediates cell division, proliferation, apoptosis and differentiation in colon epithelial cells (20). TGF−β is lost in CRC cells from early stages, thereby growth inhibition resistance is often observed. Whereas, in CRC late stages, the expression of TGF-β expression is increased leading to epithelia-to-mesenchymal transition (EMT). As a result, the normal cellular immune response was decreased due to the increased invasion and cell migration. TGF-β can also induce EMT *via* a SMAD4 independent pathway, through the Ras homolog family member A (RhoA) signaling pathway (21).

#### Notch Signaling Pathway

The Notch signaling pathway is a highly conserved intercellular pathway that regulates cell development, differentiation, proliferation, growth and apoptosis (22). The notch signaling pathway possesses 4 types of notch receptors (Notch 1-4) and 2 types of ligands (the Jagged protein family, JAG 1 and 2, and the Delta-like protein family, DLL 1, 3, and 4). The Notch receptors are transmembrane proteins containing both extracellular and intracellular domains, and Notch ligands are the single-pass transmembrane proteins containing EGF-like repeats (23). Notch signaling is initiated when Notch ligands are activated through ubiquitination by a mind bomb protein (MIB). Then, the activated ligand binds to the extracellular component of the Notch receptor and the extracellular domain of the notch receptor is cleaved by a disintegrin and metalloproteinase (ADAM protease). Subsequently, Notch intracellular domain (NICD) is cleaved by γ-secretase, causing it to dissociate from the transmembrane domain of its receptor. Next, the free NICD translocates into the nucleus and binds to the CSL (CBF-1/suppressor hairless/LAG1) transcription factor, forming a complex with co-activators MAML (mastermind-like proteins) and a histone acetylase, p300. The p300 and the histone acetyltransferase (HAT) cause the activation of transcription factors and the transcription of notch-target genes (24, 25). Previous studies have found that the Notch receptors, Notch ligands and some downstream Notch signaling targets are overexpressed in CRC (26). A recent study has reported that Notch signaling enhanced CRC severity by regulating the cell cycle and apoptosis of p21 and p53 upregulated modulator of apoptosis (PUMA) genes (27). Therefore, inhibition of Notch signaling could be one potential treatment for CRC patients.

#### NF-κB Signaling Pathway

NF-κB is a heterodimer protein, containing 2 subunits p65 and p50 which are indispensable for NF-κB activation and nuclear translocation. NF-κB family consists of five transcription factors including Rel proto-oncogene (Rel)A/p65, RelB, c-Rel, NF-κB1 (p50/p105) and NF-κB2 (p52/p100), which are involved in several biological processes such as cell development, differentiation, cell cycle, and migration. These members function as heterodimers with interrelated arms of the NF-κB pathway (28, 29). Extracellular factors such as growth factors, virus, cytokines, lipopolysaccharides (LPS), Toll-like receptor (TLR), and T/B cell receptor bind to their specific ligands resulting in an upregulation of the IκB kinase (IKK) complex. The IKK complex phosphorylates IκB which binds to p65/p50 dimers. The phosphorylated IκB is subsequently degraded by the ubiquitin-proteasome, allowing for the activation of NF-κB. Activated NF-κB is translocated to the nucleus, triggering downstream genes expression to promote CRC initiation and progression (29). On the other hand, the NF-κB pathway is also activated by ligands such as the cluster of differentiation 40 ligand (CD40L), B-cell activating factor (BAFF), receptor activator of NF-κB ligand (RANKL), lymphotoxin-β receptor (LTBR) and RelB/p100 subunits. IKKα homodimers and NF-κB-inducing kinase (NIK) are also included (30). Upon NIK activation, IKKα is further phosphorylated and induces the phosphorylation of p100, triggering the conversion of p100 to p52. The RelB/p52 dimer translocates to the nucleus to promote gene transcription. Previous studies found that NF-κB signaling is extensively implicated in CRC progression and plays important role in malignancy development in multiple stages of CRC (29). It was reported that NF-κB contributes to CRC cell growth, anchorage-independent growth and cell migration (31). Collectively, the role of NF-κB plays in CRC progression makes it’s a viable target for CRC treatment.

### CRC Microenvironment Modulation

Increasing evidence suggests that tumor progression and recurrence are not only regulated by genetic changes of tumor cells but also by tumor microenvironment (TME). TME, composed of tumor cells, stromal cells, immune cells, and extracellular matrix which surround tumor cells, is a complex system that is involved with tumor growth and development. Metastasis formation is a multistep process that promotes transformed cells to get into the bloodstream, deposited to the target organs through microvasculature circulation, and ultimately survive and grow in a foreign tissue. The immune system, as the immunosurveillance, is responsible for destroying most of the metastatic cells. With the communication of tumor cells and the cellular compartment, new cells such as inflammatory cells and immune cells are recruited into the TME, resulting in altered metabolism of surrounding stroma, and interrupted anti-tumor effect of the immune systems (32). It is known that tumor cells and TME cells can also program immune cells into tumor−tolerant or tumor−promoting phenotypes. Interleukin-6 (IL-6) is a common cytokine involved in TME and its expression is significantly elevated during CRC progression from occurrence to development. Various cells, such as tumor-associated macrophages, fibroblasts, granulocytes, dendritic cells, lymphocytes, and CRC cells are all sources of IL-6 in the TME. One study showed that tumor-associated macrophage-derived IL-6 activates the Janus kinase (JAK) 2/signal transducer and activator of transcription (STAT) 3 axis to modulate cell migration and invasion in CRC (33). Studies have also shown that IL-6 activates autophagy through the IL-6/JAK2/Beclin-1 pathway and promotes chemotherapy resistance in CRC (34).

Metastatic CRC tumors with MSI, often associated with genetic hypermutability, produce many neoantigens on the major histocompatibility complex (MHC) of antigen-presenting cells and serve as a foreigner recognized by T cells (35). Thus, the TME of CRC tumors with MSI is abundant with tumor-infiltrating lymphocytes (TILs) and the adaptive immune system plays a significant role in tumor progression suppression. Then, the strongly activated immune cells provoke the expression of immune-checkpoint receptors and ligands, such as cell surface protein programmed death-1 (PD-1) and cytotoxic T-lymphocyte-associated protein 4 (CTLA4), and transmembrane protein programmed death-ligand 1(PD-L1) on tumor cells, TILs, regulatory T (Treg) cells and tumor-associated macrophages. Therefore, as reported, patients diagnosed with metastatic CRC tumors with MSI are responsive to PD-1 blockade therapy. However, the frequency of MSI-CRC in stage IV is ~5% (36) and the majority of metastatic CRC tumors with microsatellite stable (MSS) were found to be a lower response to PD-1/PD-L1 or CTLA4 therapy (37, 38). This could be explained that CRC tumors with MSS have fewer tumor mutations and neoantigens and are lack infiltrating immune effector cells. All these factors promote tumor evasion from adaptive immunity to immune resistance against immune checkpoint therapy. Recent studies reported that in metastatic CRC tumors, a higher immune score, quantification of T cells (CD3) and cytotoxic T cells (CD8), positively correlates with a decreased metastasis (39) and can also predict the overall prolonged survival rate in patients with metastatic CRC (40).

Most recently, Osman et al. have found that T cell factor-1 (TCF-1) could control distinct clusters of Treg functions by regulating gene expression in Treg, inflammation and severity in CRC. TCF-1-deficient Treg cells strongly suppress T cell proliferation and CD8 T-cell cytotoxicity, leading to more severe and aggressive CRC (41). Although multiple transcription factors have already been well studied before this study, this is the first time that the link between TCF-1 and CRC was explored, and this pathway could help future drug development for CRC prevention or improve response to therapy.

Moreover, Cancer-associated fibroblasts (CAFs) or macrophages (CAMs) synergize the production of reactive oxygen species (ROS) which helps the tumor cells escape from the immune surveillance system (42). It is known that cancer cells can transform fibroblasts located at metastatic sites into CAFs, thus promoting tumor growth and metastasis by producing growth factors and ECM degrading proteases. Interestingly, in CRC, the release of TGF−β is correlated to redox control of TME by the activation of MAPK or ERK-mediated SMAD 2 phosphorylation, which further stimulates CAFs to secrete IL−11 and strengthen the survival ability of metastatic cells (43). CAFs have been identified as an important source of IL-6 recently and IL-6 mediated STAT3 activation in CAFs facilitates CRC tumor development (44).

What’s more, SHP2, an oncogenic tyrosine phosphatase and a major downstream signaling molecule required for the PD-1 immune checkpoint pathway, was defined to play essential roles in all cell types of TME from mouse CRC models by single-cell sequencing technology. SHP2 modulates tumor immunosuppression by negatively regulating type I interferon signaling and allosteric inhibition of SHP2 remolds the anti-tumor TME in CRC patients, indicating SHP2 is a promising target for CRC immunotherapy (45).

In summary, understanding the relationship between CRC tumors progression and microenvironment modulation could be fundamental in developing novel therapeutic strategies for a better response on CRC patients.

### CRC Epigenetic Changes

CRC progression from adenoma to adenocarcinoma is both affected by genetic and epigenetic alternations. Epigenetics is defined as heritable alterations in gene expression without DNA sequence changes, including DNA methylation, histone modification, chromatin remodeling, noncoding RNA, and microRNAs. Epigenetic disruption is a common hallmark of CRC (46). Among the above epigenetic mechanisms, DNA promoter methylation and histone modifications have gained particular interest in CRC studies because gene promoter hypermethylation transcriptionally silences the tumor suppressor genes (47) and histone modification regulates gene expression directly or by interacting with DNA methylation (48). During CRC carcinogenesis, DNA methylation typically occurs in CpG islands, characterized as the covalent modification of DNA with a methyl group (CH3) at the C5 position of the cytosine ring by DNA methyltransferases (DNMTs), generating 5-methylcytosine and this represents the most extensively studied epigenetic marks. For example, silencing of E−cadherin caused by hypermethylation results in decreased cell-cell adhesion, tumor progression and increased invasion in CRC (49).

As mentioned above, in addition to DNA methylation regulating gene expression levels, posttranslational covalent modification of histone tails is another fundamental epigenetic modification in regulating chromatin state and gene expression in human tumors. Most of the studies have focused on histone (de) acetylation, catalyzed by histone acetyltransferases (HATs) or histone deacetylases (HDACs), and methylation of lysine and arginine residues within histone tails in CRC. Histones are composed of core histones and linker histones. Specifically, the core histones consist of histones H2A, H2B, H3, and H4, and a pair of each four core histones are formed into an octamer called nucleosomes, which are interacted with other nuclear proteins to form chromatin. And the linker histones, the H1 family, localized on the entry and exit sites of the DNA to maintain its correct wrapping with core histones. Several researchers reported that global hypoacetylation in H3 and H4 lysine is often associated with decreased expression of tumor suppressors and metastasis suppressors in cell lines and primary tumors from CRC (50). It has been found that increased HDACs expression is associated with a shorter survival time of CRC patients (51). Recent studies suggested that epigenetic regulators induced transcriptional plasticity is associated with chemoresistance in CRC and these epigenetic alterations are reversible, thus providing novel opportunities for CRC treatment (52–55).

In addition, long non-coding RNAs (lncRNAs) and microRNAs (miRNAs) also play important roles in regulating signaling pathways relevant to colorectal cancer (CRC). MiRNAs function at the posttranscriptional level by regulating specific individual target mRNAs, or serve as general regulators of gene expression, simultaneously mediating hundreds of genes expression. Many studies have identified different miRNAs expression levels between neoplastic tissues and tumor-adjacent normal tissues in CRC (56, 57). MiRNAs can act both as oncogenes or tumor suppressors, depending not only on their altered pathways but also on the primary location, such as colon or rectal cancer. Specifically, miRNA-9 (58) and miRNA-101 (59) serve as tumor suppressors in CRC by suppressing colon cancer cell migration. miRNA-200 (60), miRNA-17 (61) and miR-141 (62) are all well-known oncogenes in CRC acting by inhibiting different tumor suppressor genes or promoting cancer cells proliferation. Moreover, it was reported that overexpression of miRNA-21 was correlated with the 5-Fluorouracil chemotherapeutic drug resistance in CRC and miRNA-21 reduces G2/M arrest and apoptosis by downregulating the MSH proteins. Thus, miRNA-21 could be a potential therapeutic marker in CRC (63). Despite several miRNAs having shown great value as biomarkers for disease detection (64), progression (65), and prognosis (66) in CRC, there still are several practical problems for miRNA acting as biomarkers. For example, more sensitive detection methods and lower detection costs are required (67). Moreover, miRNAs are usually not specific to one type of cancer. For example, miRNA-155 decelerates CRC metastasis and progression (68), while it serves as a potential biomarker for cervical cancer and breast cancer (69, 70). LncRNAs are involved in many CRC-related signaling pathways such as the Wnt/β-catenin, EGFR, and TGF-β, thus affecting all pathophysiological steps in CRC carcinogenesis, progression, and metastasis (71).

Overall, epigenetic changes of CRC are of great significance to early diagnosis and prognosis evaluation, providing a new thought for the CRC treatment.

### CRC and Inflammatory Bowel Disease (IBD)

Emerging evidence indicates that IBD is associated with an increased incidence of CRC development. Unlike common sCRC, IBD-CRC initiates and drives tumorigenesis from a different mechanism (72). The tumor tissues of IBD-CRC patients present less frequent somatic mutations of APC and KRAS, while p53 genomic alterations are more frequent and detected earlier during tumor progression, compared to sCRC (73–75). It has been reported that chronic inflammation in IBD promotes aberrant DNA methylation, which in turn facilitates tumor development (76). Progressively increased percentage of methylated genes in the Wnt/β-catenin pathway was observed from normal colon samples to IBD to IBD-CRC, suggesting their potential role during CRC development (75).

As previously described, tumor-associated macrophages are important immune cells in TME, and they might also play critical roles in IBD-CRC progression. It has been found that Wnt5a, a protein from the Wnt family, stimulates M2 polarization of tumor-associated macrophages *via* IL-10 to promote CRC progression (77). Thus, the Wnt/β-catenin signaling pathway can significantly impact inflammation and the IBD-CRC onset. In addition, altered M2 macrophage polarization resulting in delayed tumor progression was reported in a mouse IBD-CRC model recently (78). It is known that the two most well studies proinflammatory pathways in IBD-CRC, NF-κB and IL-6/STAT3 signaling pathways, are dysregulated and thus promote IBD-CRC progression (79). Hence, therapies against specific inflammatory cytokines involved in tumorigenesis of IBD-CRC could provide a novel approach to prevent CRC tumor initiation or progression.

### CRC Biomarkers

CRC Biomarkers, derived from a patients’ tissue, blood or stool samples, play crucial roles in the early diagnosis and prognostic stratification of the disease under targeted CRC treatment (30). Based on clinical criteria, CRC biomarkers can be divided into two groups: diagnostic biomarkers (for detection or confirmation of the presence of a disease) and clinical biomarkers (for prediction of patients’ response to a specific treatment or their prognosis).

#### CRC Diagnostic Biomarkers

It has been reported that CRC cells consistently express cytokeratin 20 and CDX-2 (an intestinal epithelia-specific nuclear transcription factor), but cytokeratin 7 expression is generally negative, therefore, these three markers can serve as the diagnostic biomarkers for CRC (80–82). Several blood-based biomarkers, such as cancer antigen 19-9, serum tissue polypeptide-specific antigen (TPS) and tissue polypeptide antigen (TPA), cytokeratin 8, 18 and 19, as well as Kininogen-1 (KNG1) were all identified as potentially useful biomarkers for both early detection and prognostic purposes in CRC (83, 84). Besides peripheral blood, cell-free DNA (cfDNA) is also used to determine cellular apoptosis in patients from CRC (85). Stool-based testing, for example, hemoglobin testing, DNA and RNA-based testing and miRNA testing have also been determined as feasible biomarkers for CRC (86–88). Researchers also reported that gene expression and metabolomic profiles of urine and tissue samples from CRC-bearing mice and CRC patients revealed metabolites associated with specific metabolic changes can indicate CRC development in early-stage and these urine and tissue biomarkers could be used in the early detection of CRC (89). Recently, a single protein marker, tissue inhibitor of metalloproteinase-1 (TIMP-1) was found to be a useful non-invasive screening marker for clinical CRC as it exhibits a potential diagnostic value with around 65% sensitivity and 95% specificity for CRC (90). What’s more, increased levels of insulin-like growth factor-binding protein 2 (IGFBP2) and pyruvate kinase (PKM2) have been revealed in CRC, appearing as diagnostic tools for early detection and screening of CRC (91).

#### CRC Prognostic Biomarkers

A CRC prognostic biomarker is defined as a marker that can be used to provide information about the outcome in CRC. Currently, the CRC staging is guided by the tumor (tumor invasion depth), node (nodal involvement) and metastasis (TNM) system. However, the prognosis outcome can be different among patients in the same disease stage. Sometimes, patients at early stages may exhibit poorer outcomes compared to patients at latter stages since CRC is a complex process with a combination of clinical and pathological variables (92). Carcinoembryonic antigen (CEA), a glycoprotein highly expressed in colorectal malignancies, is the main prognostic blood-based biomarker widely used in clinical. The elevated level of CEA positively correlates with cancer progression and indicates CRC recurrence after surgical resection. However, CEA is not specific to CRC as increased CEA levels were also observed in other complications, such as inflammatory bowel disease, hepatic metastasis, and pancreatitis (93–95). Therefore, additional prognostic biomarkers are urgently needed for CRC patients’ management and follow-up. A recent study has demonstrated that collagen proteins could be promising biomarkers for CRC metastasis through a mass spectrum-based proteomic approach, as 19 of 22 collagen alpha chains were found to be upregulated in CRC liver metastasis tissue compared to healthy adjacent liver. The upregulation of collagen type XII in the metastatic tissue was confirmed by immunohistochemistry (96). Interestingly, some studies reported that a urinary prostaglandin metabolite PGE-M might be an interesting CRC biomarker because PGE-M plays an important role in the regulation of cyclooxygenase-2 effects in CRC and elevated levels of PGE-M is associated with advanced adenomas and increased risk of CRC (97). Lymph node involvement results in poor prognosis, therefore, non-invasive protein prognostic biomarkers used for nodal status determination are urgently needed. Recently, three tissue-based protein biomarkers, FXYD3, S100A11 and GSTM3 were identified as useful markers of regional lymph node metastasis in CRC *via* a proteomic approach (98). Similarly, MX1 from CRC tissue of lymph node was also identified as a protein biomarker for predicting regional lymph node metastasis (99). Recurrence detection is most concerned for CRC patients after surgery. After surgery, around 30-40% of patients showed distant metastasis or locoregional recurrence (100). The expression of Maspin has been recently identified as a marker for early recurrence in stage IV CRC and late recurrence after surgery for CRC liver metastasis (101).

#### CRC Predictive Biomarkers

A CRC predictive biomarker is used to indicate the response of a specific treatment and is crucial for the management of CRC patients. Numerous numbers of immunotherapy, chemotherapy and targeted therapies make it necessary to discover important biomarkers for treatment response and monitoring (102, 103). Researchers found that poly(C)-binding protein 1 (PCBP1) expression was significantly elevated in oxaliplatin resistance patients than in responsive patients, suggesting that PCBP1 is a protein predictive marker of oxaliplatin resistance in CRCs (104). Katsila et al. evaluated the response to EGFR targeted therapies in plasma from patients with metastatic CRC. It was observed that plasma level of phosphorylated-EGFR (pEGFR) was correlated with sensitivity to cetuximab therapy, suggesting that circulating pEGFR is a potential predictive treatment-response biomarker (105). The response to tyrosine kinase-targeted therapies was also evaluated by McKinley et al. through a global phosphotyrosine proteomics analysis of patients with metastatic CRC treated with dasatinib, an effective inhibitor of the Src family of tyrosine kinases with significant anti-tumor effects (106). It was found that PKCdelta is a marker of responsiveness of Src inhibition in CRC cells lines, indicating that PKCdelta could be a useful biomarker for evaluation response to dasatinib in CRC. In general, the predictive biomarkers have the same problem of poor translation to clinical use as diagnostic biomarkers and further validation of clinical studies are needed before it can be translated into useful routine practice.

#### Liquid Biopsy in CRC

Recent research has focused on diverse biomarkers including circulating tumor cells (CTCs), circulating tumor DNAs (ctDNAs), circulating tumor exosomes, and circulating tumor RNAs (such as miRNAs) (107). Therefore, the concept of liquid biopsy by using these circulating biomarkers to detect tumors released from primary to metastatic sites is generated (108). Liquid biopsy has multiple advantages in terms of noninvasiveness, fast, and easy to be sampling, compared to solid biopsy. The use of CTCs in gastrointestinal cancers showed promising results in prognostic stratification, therapeutic implications, and early diagnosis (109, 110). Recently, ExoScreen technique was used for profiling exosomes such as CD147 and CD 9 in serum from CRC patients but not in healthy individuals (110). The amount of ctDNAs was also reported to be increased progressively from early stage to metastatic CRC (111). In addition to CTCs, ctDNAs, and exosomes, ctRNAs, specifically, miRNAs, are also used as CRC markers (108, 110). The application of liquid biopsy in early diagnosis, screening, and prognosis for CRC patients in clinical practice provides a new strategy for treatment guidance and post-treatment surveillance.

Thus, early diagnosis of the disease, early surgical resection and effective response of a specific treatment always offer the best chance of cure. However, accurate, and consistent protein biomarkers for CRC use are still lacking. Therefore, identification, validation, and translation of new diagnostic, prognostic, and predictive biomarkers are important to fill the presented gaps in our knowledge.

## Opportunities for CRC Treatment

Treatments of CRC include surgical resection, chemotherapy, targeted therapy, immunotherapy, gene therapy and combination therapies.

Chemotherapeutic intervention coupled with surgery is the traditional treatment for survival rate enhancement of metastatic CRC. For decades, commonly used CRC chemotherapeutic agents include 5-Fluorouracil, Irinotecan, Oxaliplatin, Calcium folinate, Capecitabine, S-1 (Tegafur/gimeracil/oteracil), and TAS-102 (Trifluridine/Tipiracil). CRC chemotherapies are usually combinations of several of those chemotherapeutic agents, such as FOLFOX (5-Fluorouracil, Calcium folinate, Oxaliplatin); FOLFIRI (5-Fluorouracil, Calcium folinate, Irinotecan); CAPEOX (Capecitabine, Oxaliplatin); FOLFOXIRI (5-Fluorouracil, Calcium folinate, Irinotecan, Oxaliplatin) (112).

Targeted therapies can be divided into two major categories: small molecule signal transduction inhibitors and monoclonal antibodies. Small molecule inhibitors can eliminate cancer cells by specifically blocking the signaling pathways necessary for tumor growth and proliferation and they can be administered orally, with high specificity and short half-life. Monoclonal antibodies, with high specificity and long half-life, recognize tumor cells through the specific antigen and antibody interaction, which are mostly administered intravenously, without hepatic metabolism. Genomic profiling for somatic mutations detection is very important because it identifies which treatments may be effective for CRC patients. Several molecular biomarkers such as KRAS mutations, BRAF mutations, and deficient MMR/MSI are commonly observed in CRC.

KRAS, a downstream pathway of EGFR, is the most observed mutation in CRC. Therefore, inhibition of EGFR can suppress the activation of KRAS and its downstream pathways. Cetuximab (Epitol), a monoclonal antibody specifically used for blocking EGFR, is one of the most representative drugs of targeted therapy and is currently approved for first-line monotherapy in CRC patients (113). However, CRC patients with KRAS mutation are very unlikely to benefit from anti-EGFR therapy. Eventually, its downstream pathways such as BRAF, MEK and ERK were continuously activated by KRAS, resulting in tumor cells proliferation and metastasis. Thus, CRC patients with KRAS mutations need to be treated with other effective therapies. Cetuximab can also be used in combination with Irinotecan, 5-Fluorouracil, and calcium folinate in the first-line treatment regimen for CRC. It was reported that the overall survival (OS) of CRC patients with wild type KRAS under Cetuximab and FOLFIRI combination treatment was improved 8.2 months compared with patients treated with FOLFIRI alone in the CRYSTAL study (114, 115). EGFR can initiate cell proliferation downstream pathway in addition to activation of KRAS. Particularly, most patients with advanced CRC are accompanied by EGFR overexpression and metastasis in the liver and lung, resulting in poor prognosis outcomes with first- and second-line therapies. Currently, clinical studies have confirmed the beneficial effects of third-line treatment of metastatic CRC with Cetuximab in Australia and Europe (116, 117).

Angiogenesis plays an important role in CRC progression and vascular endothelial growth factor (VEGF) is a major angiogenic factor in CRC (118). Increased VEGF levels were significantly correlated with CRC progression and metastasis (119). Bevacizumab, a humanized IgG monoclonal antibody, was approved by FDA as the first VEGF-targeted agent for metastatic CRC (120) and it is still the only FDA-approved VEGF-targeted agent used as a first- and second-line for CRC treatment (121). What’s more, various approved novel anti-VEGF receptor (VEGFR) agents such as Aflibercept (a VEGFR-1 and VEGFR-2 extracellular domain recombinant fusion protein) and Ramucirumab (a fully humanized monoclonal VEGFR-2-targeted IgG antibody) remained as second-line treatment of metastatic CRC. This is mainly because the combination of Aflibercept or Ramucirumab with FOLFOX regimen did not show beneficial results in progression-free survival (PFS) or response rate (122, 123). Other agents, such as various tyrosine kinase inhibitors, including Regorafenib (124) and Fruquintinib (125) are recommended for chemotherapy against refractory metastatic CRC.

Growing evidence indicates that the hepatocyte growth factor (HGF) and its tyrosine kinase receptor, mesenchymal-epithelial transition factor (c-MET) pathway plays a crucial role in tumor invasive growth and metastasis of CRC (126, 127). MET amplification and HGF/c-MET overexpression have been reported as causing factors for CRC carcinogenesis. This aberrant signaling axis triggers a series of signaling cascades activation, including EGFR/MAPK, PI3K/AKT, and JAK/STAT pathways, and is associated with dysregulated cell proliferation, apoptosis, and poor prognosis (121). Thus, various newly developed monoclonal antibodies, small molecules or miRNAs targeting HGF/c-MET have emerged. Recently, a few monoclonal HGF neutralizing antibodies have been investigated under clinical trials. Rilotumumab, a fully humanized monoclonal antibody, has demonstrated median prolonged PFS and OS in patients with MET overexpression using Rilotumumab in combination with ECX (epirubicin, cisplatin, and capecitabine) compared with those in the placebo plus ECX arm (128). There are also several novel MET antibodies, like ABT-700 and Emibetuzumab, that have been used in clinical trials (121). Small molecule c-Met kinase inhibitors target activation sites of the receptor inside the cells *via* inhibiting phosphorylation and its downstream signaling pathway. PHA-665752, c-Met inhibitor, with the combination of cetuximab suppressed more efficiently the CRC cell growth *in vitro* and *in vivo* compared with either single agent treatment (129). In addition, miRNA, such as miR-206, has been identified to be able to affect the c-MET/HGF signaling pathway *via* inhibiting CRC cells proliferation and invasion (130).

In CRC, in addition to common mutations in KRAS and BRAF, mutations in human epidermal growth factor receptor (HER) 2 are also present. Indeed, HER2 amplification has been detected in approximately 2-3% of CRC patients. However, there was no approved targeted therapy in HER2-positive metastatic CRC patients. Most recently, an open-label, multicenter, phase 2 study confirms the role of Enhertu (DS-8201, an antibody-drug conjugate targeting HER2) in HER2 overexpressing CRC. It has demonstrated that Enhertu showed promising activity in terms of objective response rate, disease control rate, PFS and OS with long-term follow-up in CRC patients with HER2 expressing which brings new hope to the majority of these patients (DS8201-A-J203, NCT03384940).

Immunotherapy, as an innovative type of cancer treatment, fights tumors by activating the body’s immune system without direct attacking tumor cells. Immune checkpoint inhibitors exhibit an immense breakthrough in cancer therapeutics and the most representative ones are PD-1/PD-L1 inhibitors and CTLA-4 inhibitors. Some of these immune checkpoint inhibitors have been approved for CRC treatment. For example, nivolumab and pembrolizumab, the two most promising PD-1 inhibitors, have been approved by FDA for the second-line treatment of metastatic CRC patients with high levels of MSI and deficient MMR in 2017 (131). However, a recent clinical study reported limited response with antibodies blocking PD1 and CTLA4 in CRC patients with MMR-MSI mutations (132). Therefore, other alternative approaches of immune modulations are required to enhance the recruitment of immune cells to the tumor, such as combination therapy. In addition to pro-proliferative effects on tumors, activation of the RAS/MAPK/MEK pathway has been associated with decreased T cell infiltration into CRC tumors and MEK is a transducer of this signaling cascade and plays a crucial role in CRC development and progression. Thus, several clinical trials with the combination of MEK inhibitors with PD-1/PD-L1 and chemotherapies are ongoing.

Gene therapy has shown remarkable efficacy in recent clinical studies (133). Lack of efficient and reliable delivery methods is the most common challenge for gene therapy, although retrovirus delivery systems are the most used. Recently, nanotechnology-based gene therapy has been approved in the clinic because it can be well-controlled (134). A group found that cancer-targeted gene therapy pigment epithelium-derived factor DNA liposomes (R-LP/PEDF) demonstrated enhanced inhibitory effects on migration, invasion, and pro-apoptosis of CRC cells and without any toxic pathological changes in the vital organs of mice that received the R-LP/PEDF treatment (135). What’s more, a nano vector made from ginger-derived lipids was reported that can serve as a delivery platform for the therapeutic agent doxorubicin to treat CRC (136). This study expands our current understanding of drug-delivery systems and could be used as a foundation for a less toxic drug-delivery approach.

In recent years, proteolysis targeting chimeric (PROTAC) technology that degrades endogenous disease-related proteins through the ubiquitin-proteasome system has achieved remarkable efficacy in tumor growth inhibition and is also a promising strategy for the drug-resistant target. As discussed previously, KRAS is a well-known “undruggable” target in CRC due to its lack of deep pockets and is associated with poor prognosis and drug resistance. It has been reported that potent KRAS-specific degraders can target tumors with mutant KRAS (137). A research group led by Professor Craig M. Crews, a pioneer of PROTACs technology, firstly reported the development of endogenous KRASG12C degrader LC-2, where over half of KRAS mutant lung cancer and 3% of CRC express KRASG12C. It was demonstrated that LC-2 rapidly and consistently degraded KRASG12C, leading to inhibition of MAPK signaling. Suggesting that PROTACs-mediated degradation is a feasible strategy to attenuate oncogenic KRAS levels and downstream signaling in cancer cells (138). Therefore, PROTAC approaches can broaden drug target scope, overcome drug resistance, enhance target selectivity and this technology could be a key therapeutic modality that provides innovative treatment options for cancer patients.

It should be noted that patients with colon and rectal cancer are managed differently, and their epidemiology and clinical outcomes also exhibit different patterns (139). Zhang et al. recently reported that several genes can be used as biomarkers to identify colon cancer from rectal cancer based on the prediction of vector machine method algorithm and RNA-sequencing data, while additional research is required to support this finding (140). However, a study conducted by The Cancer Genome Atlas (TCGA) showed similar patterns of genetic alterations in the colon and rectal tissues from non-hypermutated tumors. There were almost indistinguishable differences in copy number, CIMP, mRNA and miRNA between the colon and rectal cancers from the above samples (141). A follow-up proteomics study of colon and rectal tumors analyzed previously by TCGA identified that CRC subtypes are similar to those detected by transcriptome analysis (142). The main difference between colon cancer and rectal cancer is the anatomical location, which determines the differences in their treatments modalities such as differences in surgical approaches, and preoperative and postoperative adjuvant therapies (radiotherapies). Drugs approved for colon and rectal cancer are identical according to their identical biology. Nevertheless, chemotherapies were used differently for different purposes and indications caused by their different anatomical location (143–145).

## Conclusion

In summary, as discussed in this review, CRC represents a complex and heterogeneous group of disorders at molecular levels and signaling pathways, in which different patterns of genetic mutations and epigenetic alterations contribute to the onset and progression and are responsible for responding to the specific therapy. CRC results from the activation of multiple signaling pathways and cannot be targeted with a single treatment. Thus, a combination of conventional therapeutics with innovative inhibitors targeting different dysregulated pathways is urgently needed. A deeper understanding of CRC is required, and the efficiency of targeted therapies and the development of more efficient biomarkers provides an encouraging prospect for the future management of CRC. We believed that with the discovery of more novel targeted therapeutics, the disease burden of CRC can be decreased in the future.

## Author Contributions

ZH and MY wrote different sections of the manuscript. MY revised, wrote, and prepared the manuscript. All authors contributed to the article and approved the submitted version.

## Funding

This work was supported by Natural Science Foundation of Liaoning Province (20170541001).

## Conflict of Interest

The authors declare that the research was conducted in the absence of any commercial or financial relationships that could be construed as a potential conflict of interest.

## Publisher’s Note

All claims expressed in this article are solely those of the authors and do not necessarily represent those of their affiliated organizations, or those of the publisher, the editors and the reviewers. Any product that may be evaluated in this article, or claim that may be made by its manufacturer, is not guaranteed or endorsed by the publisher.
